# Simple ideas to mitigate the impacts of the COVID-19 epidemic on refugees with chronic diseases

**DOI:** 10.1186/s13031-020-00277-x

**Published:** 2020-05-06

**Authors:** Muhammad Fawad, Fatima Rawashdeh, Parveen K. Parmar, Ruwan Ratnayake

**Affiliations:** 1 International Rescue Committee, Khalil Dabbas St, Amman, Jordan; 2 Division of Global Emergency Medicine, Department of Clinical Emergency Medicine, University of Southern California, Los Angeles, CA 90033 USA

## Main text

Disease control amongst refugee populations is a critical component of the global response with COVID-19 [1]. Another critical issue for refugees is the maintenance of life-saving care for non-communicable and chronic infectious diseases, including diabetes and HIV/AIDS. In Jordan, Syrian refugees with non-communicable diseases typically access clinical care and medications at no cost through clinics supported by humanitarian organizations. Since January, the International Rescue Committee in Jordan has been conducting a study on the impact of supplementing clinical care with out-of-clinic support through a Syrian community health volunteer network, on adherence to medications and prevention of severe outcomes among Syrians and uninsured Jordanians with poorly controlled diabetes and/or hypertension (ClinicalTrials.gov, NCT04229667). It is notable that these patients (elderly, with multi-morbidities, and tenuous access to care), are also a major risk group for poor outcomes from COVID-19 infection [2].

On March 21, 2020, the Government of Jordan implemented national curfews to impel social distancing, including the temporary closure of clinics for refugees [3]. As clinical care is resumed, it is important to think about how to use community health volunteers to mitigate the impact of COVID-19 in this population. First, using clinic lists of diabetics and hypertensives, CHVs could help patients connect with pharmacies to ensure access to a two-month supply of medication and insulin and provide monthly follow-up by telephone for monitoring and assessment for complications from pre-existing disease. This would serve dually as infection prevention for elderly patients, minimizing their potential exposure to infected persons at primary care clinics, and would mitigate the impact of interruptions to life-saving treatment [4]. Second, community health volunteers could provide refugees with a trusted connection to surveillance systems, risk communication, and the epidemic response, which is a major aim of community engagement [5].

Such “switching-on” of remote strategies to reach patients cut-off from care is common but less documented in the literature. This has included providing “run-away” bags of antiretrovirals and a hotline for HIV/AIDS patients, and using community health volunteers to refer malnourished children and provide counseling on infant and young child feeding during deteriorations in security in Yemen [6, 7]. As with acute conflict, for COVID-19, the decision to use CHVs or other health workers to reach households is critically-dependent on operating safely (e.g. not entering households and using protection), defining a necessarily-reductive role without excessive duties, providing a critical link with health services, and securing the trust of a willing workforce. Ministries of Health and humanitarian organizations are likely thinking of such strategies for reaching vulnerable populations. We should support them with technical guidance.

## Data Availability

Not applicable.
